# The Use of Iron-Enriched Yeast for the Production of Flatbread

**DOI:** 10.3390/molecules26175204

**Published:** 2021-08-27

**Authors:** Karolina Nowosad, Monika Sujka

**Affiliations:** Department of Analysis and Evaluation of Food Quality, Faculty of Food Sciences and Biotechnology, University of Life Sciences in Lublin, Skromna 8, 20-704 Lublin, Poland; karo.nowosad@gmail.com

**Keywords:** iron deficiency, PEF, flatbread

## Abstract

The most common cause of iron deficiency is an improperly balanced diet, in which the body’s need for iron cannot be met by absorption of this element from food. Targeted iron supplementation and food fortification may be the main treatments for iron deficiency in the population. However, many iron-rich supplements and foods have low bioavailability of this element. In our study, we used yeast enriched with iron ions to produce flatbread. The yeast cells accumulated iron ions from the medium supplemented with Fe(NO_3_)_3_·9H_2_O, additionally one of the cultures was treated with pulsed electric field in order to increase the accumulation. The potential bioavailability of iron from flatbread containing 385.8 ± 4.12 mg of iron in 100 g dry mass was 10.83 ± 0.94%. All the flatbreads had a moderate glycemic index. There were no significant differences in antioxidant activity against DPPH^•^ between flatbread with iron-enriched and non-iron-enriched yeast. Sensory evaluation showed that this product is acceptable to consumers since no metallic aftertaste was detected. Iron enriched flatbread can potentially be an alternative to dietary supplements in iron deficiency states.

## 1. Introduction

Iron deficiency is a global health problem that affects people of all ages. This condition can also accompany many diseases. The etiology of iron deficiency is variable and depends on many factors that reduce iron absorption and increase the demand for this element [1]. Increased demand is a physiological condition and is commonly observed in infants, preschool children, spikes in adolescents during adolescence, and in pregnant women (mainly in the second and third trimesters) [2]. Reduced iron intake may be a direct consequence of malnutrition such as that of children and pregnant women in poor countries, or it may be attributed to a vegan or vegetarian diet that is low in iron [3]. Reduced iron absorption occurs in the presence of inhibitors such as calcium, phytates (present in grains), and tannins (present in tea and coffee). In addition, this problem also occurs after surgery that increases the pH of the stomach, which reduces conversion to ferrous ions. Diseases such as *Helicobacter pylori* infection, celiac disease and intestinal inflammation also cause reduced iron absorption [4].

Iron performs many important functions in the human body. Its primary role is to participate in the transport of oxygen through erythropoiesis, therefore, in patients with chronic inflammation, iron deficiency may be particularly severe and may exacerbate the disease state [5]. Additionally, iron deficiency is a common cause of anemia since iron is an integral part of the blood protein-hemoglobin (Hb) [6].

One way to treat iron deficiency is through oral supplementation. However, the use of dietary supplements is associated with the risk of side effects, the most common of which are gastrointestinal symptoms: Nausea, vomiting, abdominal pain, constipation, flatulence, diarrhea, occurring in up to 40% of patients [7]. Additionally, these preparations are characterized by low absorption of iron in the intestines and can have a metallic taste. For these reasons, there is a need for an effective, long-term strategic approach. In this context, food fortification with iron remains a promising and cost-effective approach to treating iron deficiency [6].

Prevention or treatment of iron deficiency can be achieved by enriching microorganisms (for example, yeast) used in the food industry with iron ions through the application of pulsed electric field (PEF) [8]. Previous studies have shown that using this technique increases the efficiency of ion accumulation by yeast from the medium [9,10,11,12,13,14]. Higher ion accumulation in cells results from the increased permeability of the cell membrane due to the phenomenon of electroporation. Electroporation consists of the development of structural defects in lipid bilayer membranes caused by the externally applied PEF [15]. It has been hypothesized that the defects are in the form of metastable nanoscale pores through which small molecules and ions could pass [15]. Metal ions adsorbed on the cell’s surface may next be a subject of intracellular bioaccumulation. This way yeasts produce metal-protein complexes called metalloproteins (or bioplexes), which are highly absorbed by the human body [16,17].The yeast biomass enriched with iron using PEF could be used for the production of functional food. 

Therefore, in our research, we produced flatbread with the addition of yeast enriched with iron ions using two methods: Only by adding iron salt to the nutrient medium and additionally supporting the accumulation by the action of pulsed electric field. Our main goal was to investigate the potential bioavailability of iron from such prepared flatbread and to examine the nutritional and antioxidant properties of this food product.

## 2. Results and Discussion

### 2.1. Nutrient Composition and Glycemic Index of Flatbreads

Table 1 presents the nutritional value and glycemic index of flatbreads produced with the addition of yeast from the cultures not supplemented and supplemented with iron by two different methods. The nutritional value of the flatbread depends mainly on the chemical composition of the flour and other ingredients used in its preparation [18]. Carbohydrates were in the range 58–62% and constituted the highest content in all analyzed samples. Samples did not differ significantly in terms of protein and fat contents.

The flatbread samples differed significantly in the ash content, which was caused by the higher content of iron in the samples enriched with this element. The amount of carbohydrates, fats, and proteins influenced the caloric value of the products. There were statistically significant differences in the caloric value of the flatbreads, those with the addition of control yeast C1 had the highest caloric value, and the flatbreads with yeast enriched with iron using PEF had the lowest value.

Grain products, apart from fruit and vegetables, are the basis of the human diet. Flatbread is the oldest form of food that is still widely consumed in the Middle East, and due to the composition and methods of preparation, several varieties are distinguished, e.g., chapatti, lavash or tortillas [19]. The glycemic index (GI) is an index of foods that contain carbohydrates. It classifies foods based on their postprandial glycemic response against a reference carbohydrate source (glucose or white bread). It ranges from 1–100 [20]. The IG value depends on the size of starch molecules and the ratio of amylose to amylopectin, as well as the content of protein, fat, fiber, anti-nutrients, and organic acids [21]. The investigated flatbreads did not differ significantly in IG and the values obtained in our study were mainly influenced by the type of flour used (refined wheat flour). However, these GI values were lower than those reported in the literature for traditional Indian flatbread [22] and can be classified as a product with a moderate GI.

### 2.2. Color Measurements

Table 2 shows the color of the flatbread surfaces in terms of L*, a*, b*, and ΔE values. The higher content of iron ions in the sample resulted in a decrease in brightness (L*) and yellowness (b*), increased redness (a*), thus an increase in the ΔE value for the sample with yeast P. This is the expected effect as the color of the baked goods depends on the color of the raw materials used. The iron-enriched yeast (C2 and P) were clearly darker than the unenriched yeast (C1). However, changing the color of iron-containing products may increase their acceptance, since darker products are perceived by consumers as healthier and associated with a higher content of health-promoting ingredients, e.g., dietary fiber [23].

### 2.3. The Potential Bioavailability of Iron

Bioavailability is defined as the ability of a nutrient to be released from the food matrix and dissolved. It determines the amount of the active substance that enters the systemic circulation from the administered dose, as well as the rate of absorption of this substance. Many factors influence the bioavailability of a substance. It largely depends on the disruption of the permeability of the natural or processed food matrix, which in turn leads to the release of the nutrient that is absorbed in the gastrointestinal tract [24]. Iron absorption inhibitors are, for example, phytates, i.e., salts of phytic acid found in plants; polyphenols present in vegetables, fruits, some grains and legumes, tea, coffee, and wine. Calcium has also been shown to negatively affect non-heme and heme iron absorption, differentiating it from other inhibitors that only affect non-heme iron absorption [25]. One of the ingredients that improves iron absorption is ascorbic acid. This effect is largely due to its ability to reduce iron (III) to iron (II) as well as its ability to chelate iron [26]. In our study, refined wheat flour was used to produce the flatbread. The study did not analyze the content of phytate in flour and its influence on the bioavailability of iron. However, literature data show that similar flours are characterized by a phytate concentration of ≈100 mg/100g of flour. The effect of phytic acid on the bioavailability of non-heme iron is well known [27]. Since the same flour was used to obtain all the flatbreads and in the same proportion, the effect of phytates on the bioavailability of iron was ignored.

Table 3 presents the iron content and potential bioavailability of this metal from flatbreads. The flatbread obtained with the addition of unenriched yeast contained only about 3 mg/100 g dry mass of iron, and that with the enriched yeast, but without PEF, obtained about 266 mg/100 g of dry mass. The use of iron-enriched yeast in PEF conditions for the preparation of dough increased the iron content in the flatbreads to almost 386 mg/100 g dry mass. There was a significant difference in the potential bioavailability of iron between the samples. The flatbread with yeast P had the highest iron bioavailability, which was correlated with the highest content of this element in the product.

Many studies have been done to evaluate the iron bioavailability of cereal products fortified with iron [28]. However, it is difficult to compare the results due to differences in research methodology. For example, Pizarro et al. [26] prepared bread enriched with ferric sulphate which contained 47 mg of this element in 1 kg. The authors reported that the average iron absorption from this product determined in vivo was 10.5%, which is comparable to our results. There is a lack of reports using iron-enriched yeast for preparation of cereal products. In a study by Sabatier et al. [29], iron-enriched yeast were used for fortification of cheese. The authors determined the bioavailability of iron, but they used an in vivo method so it is difficult to compare the results. However, they concluded that iron from iron-enriched yeast was 72–82% as well absorbed as ferrous sulfate. Additionally, this study showed that during gastric and intestinal digestion in vitro, yeasts are lysed and release most of the iron after 1–3 h of the process. In our previous studies, we have shown that iron ions are bound by functional groups present both in the cell wall and in the intracellular structures of yeast [30].

### 2.4. Antioxidant Activity of Flatbread

**Figure 1:**
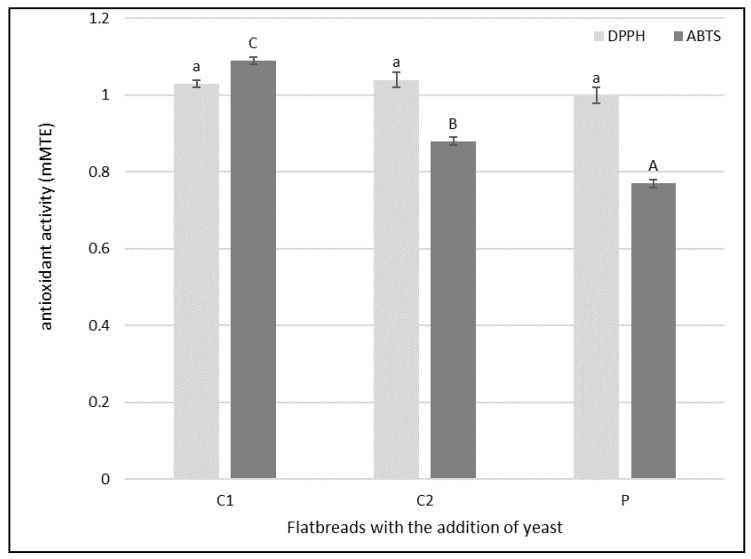
Antioxidant properties of flatbread produced with yeast not enriched with iron ions (C1), with yeast enriched with iron ions without PEF(C2), and with yeast enriched with iron ions using PEF(P), expressed as Trolox equivalent antioxidant activity. Each value is the mean ± standard deviation (*n* = 3). Bars with the same letter are not significantly different (*p* < 0.05).

Measuring the antioxidant properties of food products provides information about the antioxidant activity of a given product that may occur in the human body. The antioxidant activity of the flatbread was tested by the ability of the extracts to inhibit DPPH^•^ and ABTS^•+^. The investigated properties are presented in Figure 1. In the case of antioxidant activity against DPPH^•^ no significant differences were found between the samples. The highest values of antiradical activity against ABTS^•+^ were determined in flatbread with yeast C1, and the lowest in flatbread with yeast P (1.09 and 0.77 mMTE, respectively). The lower antioxidant capacity of the extract from flatbread with yeast P may result from the nature of iron, as iron is a metal with redox activity that can participate in electron transfer reactions, which in turn causes the production of oxidants capable of oxidizing cell components. Iron can participate in the catalysis of the formation of highly reactive hydroxyl radicals from hydrogen peroxide (H_2_O_2_) in the Haber-Weiss reaction and decompose lipid peroxides into peroxy and alkoxy radicals, which promotes lipid oxidation [31].

### 2.5. Sensory Evaluation

The results of the quality assessment of the flatbread using the 5-point rating scale are presented in Table 4. The quality of all the flatbreads was rated good and flatbread produced with yeast P obtained the highest average score. What is important, the panelist did not perceive a metallic after-taste which is a serious problem for products enriched with iron salts. The accumulation of iron in yeast may reduce unfavorable changes in taste in food products, since metal ions are associated with the cell organelles [30].

The consumer’s acceptance of the food products depends mainly on their sensory characteristics and health promoting properties. Due to the growing awareness of a healthy lifestyle, the importance of cereal products that contain whole grain or other functional ingredients is also increasing [18]. 

The use of iron-enriched yeast as a supplement is becoming more and more popular [32,33,34]. However, there are still few reports of the use of iron-enriched yeast in food products.

Iron compounds that are characterized by poor solubility at normal gastric acid concentrations do not interfere with the sensory properties of food [35]. On the other hand, more soluble compounds can cause fat oxidation (i.e., going rancid) as well as a color change in the product within 6 months of storage. Reduced iron is preferred, and the smaller the particle size, the better it will be absorbed [36]. According to Kiskini et al. [37], who compared sensory characteristics of unenriched bread with breads that were enriched with various iron compounds, ferric pyrophosphate was the compound that after addition to bread gave the most acceptable product, while bread with ferrous lactate was the least acceptable. However, all the products had a pungent odor that was not found in the iron-enriched flatbread obtained in this study.

The sensations of smell and taste consist of the basic smell and taste derived from the aromatic compounds of the basic ingredients and aromatic additives in the composition of the raw materials. The smell of a product will meet the required standards if it is characteristic for a given product and will be free of foreign, unusual smells [38]. According to the established average values of the smell of the flatbread, the highest score of 0.39 was given to the flatbread with yeast C1 (unenriched with iron), but the differences between the scores for all the flatbreads were very small.

Appearance is based on the sense of sight and includes an ability that can be examined visually. Consumers often decide whether to buy a given product based on the appearance of the product. Color also affects the appearance. This is an important feature as it is used as a control parameter when baking wheat flour products [38]. It can be concluded from Table 4 that the color of the flatbread with the addition of C1 and P yeast was ranked the highest. The color of the flatbread with the addition of P yeast resembled whole grain products, which, due to the growing awareness of consumers about a healthy lifestyle, are more acceptable and desirable.

The flatbreads with the addition of yeast P obtained the highest scores for structure and consistency. The quality of flatbread is primarily influenced by the type of flour used and the total protein content in the flour. Many of the quality characteristics, e.g., external appearance, structure, and consistency, depend on the quality and quantity of the protein. In leavened bread, the higher water absorption results in more carbon dioxide bubbles and a coarser structure of the bread. The water used when mixing the ingredients allows the formation of gluten as a result of protein hydration and the change of rheological properties [18].

### 2.6. Limitations and Future Perspectives

The results obtained in this study suggest that the use of yeast enriched with iron through its accumulation enhanced by PEF is a promising tool for the production of functional foods that may be effective against, e.g., anemia. The main limitation of the use of PEF to enrich yeast with bio-elements, including iron, is that despite many scientific studies on the principles and applications of PEF published so far, this technology is still considered emerging. There are still no specific regulations in the European Union for food processed with PEF. Moreover, consumers are suspicious of food produced with the use of unconventional methods. What is more, the use of such methods entails additional costs. Therefore, at present, the fortification of food with iron-enriched yeast cannot yet compete with the fortification with iron salts in this respect.

## 3. Materials and Methods

### 3.1. Ingredients for the Production of Flatbread

Flatbread was prepared in three trials with the addition of yeast not enriched with iron ions (C1), yeast enriched with iron ions (C2) by a supplementation of medium, and yeast enriched with iron ions using a pulsed electric field (P). Additionally, 450 g of wheat flour (white wheat flour for pastry, type 450), 7 g of freeze-dried yeast (the composition of yeast is given in Table 5), salt, olive oil, and 30 mL of warm water were used to prepare the dough.

### 3.2. Yeast Strain and Culture Conditions

In the experiment, the yeast strain *Saccharomyces cerevisiae* 11 B1 from the Department of Biotechnology, Microbiology and Human Nutrition of the University of Life Sciences in Lublin, was used. Then, agar slants and inoculum were prepared according to Romani and Maguire [39].

### 3.3. Preparation of Yeast Culture

Ten milliliters of the inoculum was added to 80 mL (C2, P) or 90 mL (C1) of the culture medium in a 500 mL Erlenmeyer flask. The cultivation was carried out under the same conditions as the inoculum. To each flask (except sample C1), 10 mL of iron (III) nitrate solution was added so that the final concentration of iron ions in the medium was 200 µg Fe^2+^/mL. The culture was then incubated at 30 °C for 20 h. The culture P was exposed to PEF for 20 min at a pulse width of 10 μs, an electric field voltage of 1500 V, at a field frequency of 1 Hz using a laboratory electroporator (ECM 830, BTX Harvard Apparatus, Holliston, MA, USA)(except for control samples C1 and C2). Cultures were then incubated for 22 h. The biomass was centrifuged (10 min, 3000 rpm), the supernatant was discarded, and the cells were washed three times with deionized water [30] and freeze-dried (Labconco, Kansas City, MO, USA).

### 3.4. Preparation of Flatbread

Seven grams of yeast (C1, C2, P) was grown in warm water (30 mL) for 45 min at room temperature. Then, 450 g of flour, 1 g of salt, and 10 g of olive oil were added to the prepared mixture. The mixtures were prepared in the traditional way by weighing the ingredients according to the recipe, thorough mixing, and aeration. Afterwards, the dough was rolled out and fried in a hot pan on both sides for 2 min. At the same time, control samples were prepared for the flatbread containing yeast without the addition of iron ions (C1) and yeast with the addition of iron ions and not treated with PEF (C2). Each type of flatbread was produced in three repetitions.

### 3.5. Nutrient Composition and Energy Content

Flatbreads were analyzed for protein content by the Kjeldahl method (N × 6.25), fat, and ash using standard analyzes [40]. The carbohydrate content was calculated according to the formula: 100−(weight in grams (protein + fat + ash) in 100 g of dry weight of flatbreads.

The energy content of the products was determined by multiplying the values obtained for protein, available carbohydrates, and fat by 4.00, 4.00, and 9.00, respectively, and adding up the results [41].

### 3.6. Color Measurements

From each trial, 10 g of flat bread was randomly selected and the color was measured with an EnviSense NH310 colorimeter (EnviSense, Lublin, Poland) in triplicate. Color differences were recorded on the CIE L* a* b* scale with respect to brightness (L*) and color (a*—redness; b*—yellow). The total color difference (Δ*E*) was calculated from the formula (1):

(1) $$\Delta E=\sqrt{\Delta L^{*2}+\Delta a^{*2}+\Delta b^{*2}}$$

where ∆L*,∆a*, and ∆b* are differences in the L*, a*, and b* values, respectively, between the reference sample and the test sample.

### 3.7. Antioxidant Properties

#### 3.7.1. Extraction of Bioactive Compounds

The samples (1 g) were ground in a laboratory grinder and shaken with 10 mL of 4:1 ethanol/water (*v*/*v*) for 120 min in a laboratory shaker. Next, the samples were centrifuged at 3000 g for 10 min. The supernatant was taken and stored at −18 °C.

#### 3.7.2. DPPH Radical Scavenging Activity

The DPPH assay was performed according to Brand-Williams et al. [42] with modification. An aliquot of 0.1 mL of the sample was mixed with 0.9 mL of a 6 µM solution of DPPH^•^ in 75% methanol and left for 3 min. The absorbance at 515 nm was then measured against 75% methanol as a blank. The determination was performed in triplicate. The scavenging effect was calculated according to the formula (2):

(2) $$\mathrm{Scavenging}\;\mathrm{activity}\;\left(\%\right)=\left[1-\left(\frac{A\;\mathrm{sample}}{A\;\mathrm{control}}\right)\right]\times 100$$

where the A sample is the absorbance of the mixture of sample and DPPH^•^ and the A control is the absorbance of the control (DPPH^•^ solution). The results were expressed as Trolox equivalent antioxidant activity (TEAC) values (mM Trolox).

#### 3.7.3. ABTS Radical Scavenging Activity

The ABTS assay was performed according to Re et al. [43] with slight modifications. Here, 2.90 mL of the ABTS^•+^ solution was mixed with 0.1 mL of each sample. The absorbance was measured at 734 nm after 3 min of the reaction against deionized water. The scavenging effect was calculated using Equation (3):

(3) $$\mathrm{Scavenging}\;\mathrm{activity}\;\left(\%\right)=\left[1-\left(\frac{A\;\mathrm{sample}}{A\;\mathrm{control}}\right)\right]\times 100$$

where the A sample is the absorbance of the mixture of sample and ABTS^•+^ and the A control is the absorbance of the control (ABTS^•+^ solution).The results were expressed as Trolox equivalent antioxidant activity (TEAC) values (mM Trolox).

### 3.8. Potential Bioavailability of Iron from Flatbread

**Figure 2:**
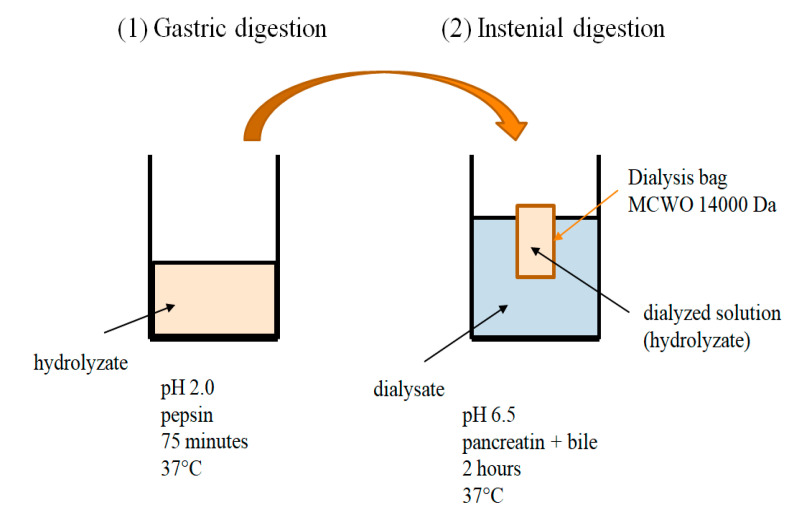
Scheme of in vitro digestion: (**1**) gastric digestion: enzyme: pepsin, pH: 2.0, digestion time: 75 minutes at 37 °C; (**2**) intestinal digestion: enzymes: pancreatin and bile, pH: 6.5, digestive time: 2 hours in 37 °C.

The in vitro digestion was performed according to Szalast-Pietrzak et al. [44] with slight modifications. Here, 1 g of the sample was combined with 30 mL of deionized water and 1 M HCl to obtain a pH of 2.0 and treated with pepsin (Sigma-Aldrich, St. Louis, MO, USA) in the amount of 2 mL of a 10% enzyme solution in 0.1 M HCl per test system. The reaction was carried out for 75 min at 37 °C with stirring (130 rpm). The second step of the in vitro process, corresponding to intestinal digestion, was performed using dialysis tubes with a molecular weight cutoff of 14 kDa. In this digestion stage, the pH of the test systems was adjusted to pH 6.5 with 6% NaHCO_3_ solution and treated with pancreatin (Sigma-Aldrich) in the amount of 5 mL of 0.4% enzyme solution in 0.1 M NaHCO_3_ per test system. Then, the samples were quantitatively transferred to dialysis tubes, which, after being sealed, were placed in laboratory containers made of PP material in 500 mL of deionized water. After the intestinal digestion step, 3 mL of dialysate and dialysis solution were taken (Figure 2).

The iron content of the dialysate, the dialysis solution, and the product before digestion was determined by the FAAS method. The data were substituted into a formula (4) taken from Szalast-Pietrzak et al. [44] and the potential bioavailability of iron was calculated:

B% = [D + Dr/(T + D)] × 100%(4)

where D is the amount of iron (mg) in the dialysate, Dr is the amount of iron (mg) contained in the tube dialysis treatment corresponding to the balance of concentrations, and T is the amount of iron (mg) in the tube (mineralizate).

The amount of iron (mg) in the dialysis tube corresponding to the equilibrium concentrations of the given test system was calculated according to the formula (5) taken from Szalast-Pietrzak et al. [44]:

Dr = (D × Vt)/Vd(5)

where D is the amount of iron (mg) in the dialysate, Vt is the sample volume in the tube (mL), and Vd is the dialysate volume (mL).

### 3.9. In Vitro Glycemic Index (GI)

The glycemic index (GI) of flatbread was determined according to the method of Reis and Abu-Ghannam [45] with slight modifications. The digestion procedure outlined in Section 3.8 was used. Here, 1 mL of the hydrolyzate was collected during the in vitro digestion period at 10, 20, 30, 60, 90, 120, and 180 min of digestion. Then, 4 mL of ethanol was added to 1 mL of the hydrolyzate to deactivate the enzymes. The glucose content in the hydrolysates was determined using the GOPOD method. Values are expressed as mg glucose/g sample. The glucose content was plotted as a function of time and the areas under the hydrolysis curves (AUC) were calculated. The hydrolysis index (HI) for each sample was calculated as the ratio between the AUC of the sample and the AUC of the reference food, which was white bread. The value was expressed as a percentage. The GI was calculated according to the Equation (6) described by Goñi, Garcia-Alonso and Saura-Calixto [46]:

GI (%) = 39.71 + 0.549 × HI(6)

### 3.10. Sensory Evaluation

A group of 15 trained people aged from 28 to 50 participated in the sensory test using a five-point rating scale with definitions for each point value on the scale. People participating in the evaluation received previously prepared evaluation cards (Table 6). The evaluation of qualitative factors such as: color, smell, structure and consistency, as well as taste was made on the basis of the developed scheme (Table 7). The individual qualitative characteristics were assigned a weighting factor. A grade of 5 meant a very good class, 4—a good class, 3—a satisfactory class, 2—an insufficient class, and 1—a bad class. The samples were codded with three-digit numbers and served on white plates. The assessments were made in the correct order. First, the visual characteristics were assessed, then the rheological ones, and the last was the taste of the product. 

### 3.11. Statistical Analysis

Regression analyses and significance tests were performed using the Statistica version 13.3 software (StatSoft, Inc., Tulsa, OK, USA). The post-hoc Tukey test was employed to determine differences between means. Results of *p* < 0.05 were considered statistically significant.

## 4. Conclusions

Micronutrient malnutrition is a major contributor to the increase in the incidence of various diseases. Iron deficiency is a common cause of anemia that affects people of all ages and around the world. Food fortification with iron compounds is one of the strategies for its prevention, but unfortunately iron can cause unacceptable sensory changes in products. In our study, rather than adding iron compounds directly to the product, we used yeast enriched with iron ions to produce flatbread. We applied pulsed electric field to enhance iron accumulation in yeast cells. The obtained flatbread contained about 386 mg/100 g dry mass of iron, had good potential bioavailability of this element, was acceptable to consumers (no metallic aftertaste was detected), and had a moderate glycemic index.

## Figures and Tables

**Table 1:** Nutritional composition, caloric value, and glycemic index (GI) of flatbreads(content given on a dry mass) produced with yeast not enriched and enriched with iron ions.

Flatbread with Yeast	Proteins (%)	Fat (%)	Carbohydrates (%)	Ash (%)	Caloric Value (kcal/100g)	Caloric Value (kJ/100g)	Glycemic Index (IG)
C1	11.85 ± 0.42 ^a^	4.50 ± 0.33 ^a^	62.09 ± 0.48 ^c^	21.56 ± 0.19 ^a^	336.23 ± 2.38 ^c^	1406.79 ± 9.97 ^c^	56.24 ± 0.12 ^a^
C2	11.44 ± 0.14 ^a^	4.81 ± 0.18 ^a^	59.42 ± 0.18 ^b^	24.33 ± 0.33 ^b^	326.70 ± 2.18 ^b^	1366.91 ± 9.11 ^b^	56.51 ± 0.25 ^a^
P	12.39 ± 0.45 ^a^	4.11 ± 0.19 ^a^	58.14 ± 0.40 ^a^	25.37 ± 0.35 ^c^	319.07 ± 2.26 ^a^	1334.97 ± 9.45 ^a^	56.23 ± 1.34 ^a^

C1—yeast without the addition of iron ions and without PEF; C2—yeast with the addition of iron ions and without PEF; P—yeast with the addition of iron ions and PEF. Each value is the mean ± standard deviation (*n* = 3). Results with the same letter within a column are not significantly different (*p* < 0.05).

**Table 2:** Color determinants of flatbread.

Flatbread with Yeast	L*	a*	b*	ΔE
C1	94.98 ± 0.54 ^c^	1.00 ± 0.18 ^a^	11.32 ± 0.52 ^b^	-
C2	93.29 ± 0.22 ^b^	1.54 ± 0.07 ^c^	12.86 ± 0.14 ^c^	3.78
P	91.53 ± 0.95 ^a^	1.23 ± 0.1 ^b^	10.47 ± 0.1 ^a^	4.43

C1—yeast without the addition of iron ions and without PEF; C2—yeast with the addition of iron ions and without PEF; P—yeast with the addition of iron ions and PEF. Each value is the mean ± standard deviation (*n* = 3). Results with the same letter within a column are not significantly different (*p* < 0.05).

**Table 3:** Iron content and potential bioavailability of iron from flatbread.

Flatbread with Yeast	Iron Content (mg) in 100 g of Dry Mass	The Potential Bioavailability of Iron (%)
C1	2.96 ± 0.54 ^a^	5.86 ± 0.12 ^a^
C2	266.3 ± 2.62 ^b^	7.97 ± 0.64 ^b^
P	385.8 ± 4.12 ^c^	10.83 ± 0.94 ^c^

C1—yeast without the addition of iron ions and without PEF; C2—yeast with the addition of iron ions and without PEF; P—yeast with the addition of iron ions and PEF. Each value is the mean ± standard deviation (*n* = 3). Results with the same letter within a column are not significantly different (*p* < 0.05).

**Table 4:** Results of the flatbread evaluation with the 5-point rating scale.

Feature	Weighting Factor	Flatbread with Yeast		
		C1	C2	P
Color	0.3	1.32 ± 0.10 ^b^	1.22 ± 0.04 ^a^	1.32 ± 0.03 ^b^
Smell	0.15	0.39 ± 0.01 ^b^	0.37 ± 0.02 ^b^	0.33 ± 0.01 ^a^
Structure and consistency	0.15	0.50 ± 0.08 ^a^	0.56 ± 0.06 ^a^	0.60 ± 0.02 ^a^
Taste	0.4	1.55 ± 0.04 ^a^	1.49 ± 0.08 ^a^	1.68 ± 0.08 ^b^
Overall		3.76 ± 0.12 ^a^	3.64 ± 0.11 ^a^	3.93 ± 0.03 ^b^

C1—yeast without the addition of iron ions and without PEF; C2—yeast with the addition of iron ions and without PEF; P—yeast with the addition of iron ions and PEF. Each value is the mean ± standard deviation (*n* = 3). Results with the same letter within a row are not significantly different (*p* < 0.05).

**Table 5:** Composition of wheat flour and freeze-dried yeast used for the production of flatbread (content given on a dry mass).

Component	Protein (%)	Carbohydrates (%)	Fat (%)	Iron Content (mg/g)
Wheat flour	10.8 ± 0.21 a	65.7 ± 0.18 c	1.35 ± 0.12 a	0.70 ± 0.01 a
Yeast C1	59.13 ± 0.18 b	31.79 ± 0.28 a	2.7 ± 0.14 b	0.13 ± 0.01 b
Yeast C2	58.24 ± 0.35 c	30.44 ± 0.59 b	2.5 ± 0.00 c	18.68 ± 0.86 c
Yeast P	54.0 ± 0.11 d	31.00 ± 0.37 b	2.35 ± 0.07 d	48.01 ± 0.88 d

C1—yeast without the addition of iron ions and without PEF; C2—yeast with the addition of iron ions and without PEF; P—yeast with the addition of iron ions and PEF. Each value is the mean ± standard deviation (*n* = 3). Results with the same letter within a column are not significantly different (*p* < 0.05).

**Table 6:** Evaluation card for the 5-point rating scale.

Feature	Sample No.		
	218	331	829
Color			
Smell			
Structure and consistency			
Taste			

**Table 7:** Scheme of the 5-point rating scale.

Feature	Weighting Factor	Definitions				
		5	4	3	2	1
Color	0.30	Light with brown baked bubbles	Light with little-baked bubbles	Bright, with traces of baked bubbles	No characteristic baked bubbles	Inappropriate color—very light or very dark
Smell	0.15	Very aromatic, typical for a baked flour product	Aromatic, typical for a baked flour product	A noticeable smell of a baked flour product	Faint smell of baked flour product, perceptible smell of a burnt product	No smell characteristic of flour products, a very perceptible smell of a burnt product, foreign smell
Structure and consistency	0.15	Very well baked, compact, uniform, easily brittle	Well baked, brittle	Sufficiently baked and hard, brittle	Poorly baked, not too hard, not brittle	Underdone, rubbery
Taste	0.40	Very natural, mild, characteristicfor flour product	Natural, gentle, desirable	Sufficiently natural, mild, without any foreign taste	Very poor taste of flour products, not very natural	No flour taste, bitter taste, metallic after-taste

## Data Availability

Not applicable.

## Sample Availability

Samples of the compounds are not available from the authors.
